# Mycetoma

**DOI:** 10.4269/ajtmh.2011.10-0637

**Published:** 2011-11-01

**Authors:** Fernanda Cordeiro, Carolina Bruno, Carmelia Reis

**Affiliations:** Dermatology Department, Brasilia's University Hospital, University of Brasilia, Brasilia, Federal District, Brazil

**Figure 1.**
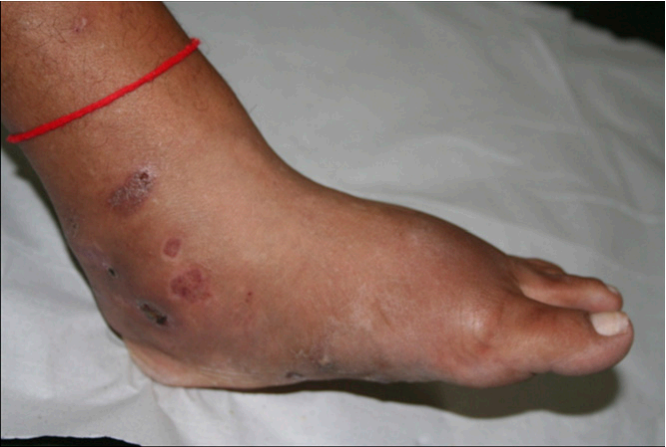
Clinical appearance of sinus tracks on medial aspect of right ankle and diffuse ankle and foot swelling suggestive of mycetoma.

**Figure 2.**
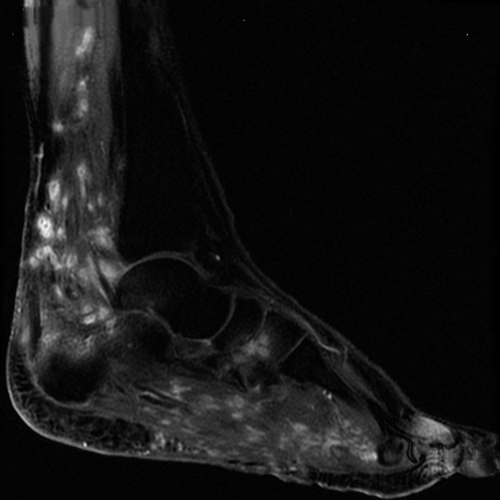
MRI correlation of right lower leg (sagittal section, post-gadolinium) showing ring-enhancing nodules and bone marrow infiltration of the calcaneus. Spherical lesions showing hyperintense signal, the so-called dot-in-circle sign in mycetoma.

A 32-year-old woman from northeastern Brazil worked in farming and presented with a 6-year history of progressive swelling of the left foot. Swelling and sinuses draining purulent discharge with white grains (Figure 1) were present. Potassium hydroxide preparation of drainage showed white-yellowish nodules with fine filaments. Culture of the grains on Sabouraud agar and the casein hydrolysis test confirmed the presence of *Nocardia brasiliensis*. Hematoxylin/eosin-stained sections showed microabscesses, granulomatous reaction, and nodules containing basophilic bacterial filaments peripherally surrounded by eosinophilic pillars. The clinical diagnosis of mycetoma was thus confirmed as a bacterial mycetoma. Magnetic resonance imaging (sagittal section, post-gadolinium) showed small nodules with intensive ring enhancement distributed in the distal left leg and foot (Figure 2) along with bone marrow infiltration of the calcaneus. Some spherical lesions showed hyperintense signal in the periphery and low-signal intensity central foci consistent with the recently described dot-in-circle sign.^1^,^2^ The patient was treated with amikacin (5 cycles with 15 mg/kg/day, for 21 days), trimethoprim sulfamethoxazole (800/160 mg, b.i.d., for 2 years), and three debridement procedures resulting in a satisfactory clinical response. Magnetic resonance imaging may be useful for the early diagnosis of mycetoma. Delayed diagnosis of this condition is common because of limitations of conventional microbiological methods that can lead to severe consequences including amputation.
